# GLT1 gene delivery based on bone marrow-derived cells ameliorates motor function and survival in a mouse model of ALS

**DOI:** 10.1038/s41598-021-92285-x

**Published:** 2021-06-17

**Authors:** Natsuko Ohashi, Tomoya Terashima, Miwako Katagi, Yuki Nakae, Junko Okano, Yoshihisa Suzuki, Hideto Kojima

**Affiliations:** 1 Department of Stem Cell Biology and Regenerative Medicine, Shiga University of Medical Science, Seta Tsukinowa-Cho, Otsu, Shiga 520-2192 Japan; 2 Department of Plastic and Reconstructive Surgery, Shiga University of Medical Science, Shiga, Japan

**Keywords:** Neurological disorders, Genetic transduction

## Abstract

Amyotrophic lateral sclerosis (ALS) is an intractable neurodegenerative disease. CD68-positive bone marrow (BM)-derived cells (BMDCs) accumulate in the pathological lesion in the SOD1(G93A) ALS mouse model after BM transplantation (BMT). Therefore, we investigated whether BMDCs can be applied as gene carriers for cell-based gene therapy by employing the accumulation of BMDCs. In ALS mice, YFP reporter signals were observed in 12–14% of white blood cells (WBCs) and in the spinal cord via transplantation of BM after lentiviral vector (LV) infection. After confirmation of gene transduction by LV with the CD68 promoter in 4–7% of WBCs and in the spinal cord of ALS mice, BM cells were infected with LVs expressing glutamate transporter (GLT) 1 that protects neurons from glutamate toxicity, driven by the CD68 promoter, which were transplanted into ALS mice. The treated mice showed improvement of motor behaviors and prolonged survival. Additionally, interleukin (IL)-1β was significantly suppressed, and IL-4, arginase 1, and FIZZ were significantly increased in the mice. These results suggested that GLT1 expression by BMDCs improved the spinal cord environment. Therefore, our gene therapy strategy may be applied to treat neurodegenerative diseases such as ALS in which BMDCs accumulate in the pathological lesion by BMT.

## Introduction

Amyotrophic lateral sclerosis (ALS) is a neurodegenerative disorder characterized by degeneration and cell death in upper and lower motor neurons. ALS results in progressive weakness and atrophy of voluntary skeletal muscles, which lead to paralysis and death due to respiratory failure in its terminal stage. Respiratory muscle dysfunction and bulbar palsy cause a major reduction in the survival of patients. Thus, the current treatment is based on symptom management and respiratory support with only a few approved medications, which provide modest benefits in some patients^1,2^. Moreover, treatment of this fatal neurodegenerative disease remains obscure because its pathophysiology is not well understood.

Many molecules have been investigated as potential treatments for ALS. For example, treatment with antioxidants and growth factors, such as insulin-like growth factor-1, hepatocyte growth factor, and glial cell line-derived neurotrophic factor (GDNF), has been used to promote neuronal protection in ALS model rodents^3–7^. Recently, regenerative treatment by stem cells has attracted attention and various cell transplantations have been evaluated^8–12^. However, although stem cell therapy has benefits, it is very important to improve the environment around neurons by growth factors or anti-inflammatory molecules^10–12^.

As a perspective of supporting the importance of the environment around neurons, the concept of “non-cell autonomous neuronal death” has received much attention in the pathophysiological condition of neurodegenerative diseases such as ALS^13,14^. In fact, microglia are related to the pathogenesis of neurodegenerative diseases by inducing focal inflammation or protecting neurons depending on the disease status^15^. Many CD68-positive cells and microglia have been observed in the spinal cords of ALS patients and ALS model mice^16,17^. In addition, CD68-positive cells had accumulated greatly in the spinal cord with the advanced disease condition of ALS^16,17^.

As a treatment strategy for ALS, bone marrow transplantation (BMT) has been performed^18^. Wild type BM cells and hematopoietic stem cells transplanted into SOD1(G93A) mice, an ALS model, have been shown to preserve motor functions and prolong the survival of the mice^18^. We have previously identified stem cell factor (SCF)-activated BM cells as a potential new therapeutic agent for the treatment of ALS^19^. A large number of CD68-positive BM-derived cells (BMDCs) were observed in the spinal cords from SOD1(G93A) mice and the number of cells was increased gradually as the disease advanced. Furthermore, pretreatment of BM cells with SCF improves the treatment efficacy through expression of glutamate transporter (GLT) 1^19^. GLT1 is a sodium-dependent transporter that plays an important role in glutamate homeostasis by removing excess glutamate in the central nervous system. GLT1 is expressed primarily in astrocytes and has been shown to be expressed in axon terminals of neurons^20–22^, and its dysregulation occurs in various neurological diseases including Huntington’s disease, Alzheimer’s disease, Parkinson’s disease, epilepsy, and ALS^23–26^. In previous reports, supplying of GLT1 is shown to be effective for the delay of disease progression of some neurodegenerative diseases^27^. Therefore, GLT1 has become an attractive target for therapeutic intervention because of its dysregulation in neurological diseases.

Here, we present a new therapeutic strategy with BMDCs-based gene delivery of GLT1 for ALS. The most notable point of this strategy is the increasing accumulation of BMDCs carrying the therapeutic gene as the pathological condition progresses, which ameliorates motor functions and survival of SOD1(G93A) mice. These results are the proof-of-principle that our gene therapeutic strategy may be used to treat neurodegenerative diseases such as ALS, in which BMDCs accumulate in the pathological lesion by BMT.

## Results

### Gene transduction efficiency in BM cells after lentiviral infection and BMT of wild type mice

**Figure 1:**
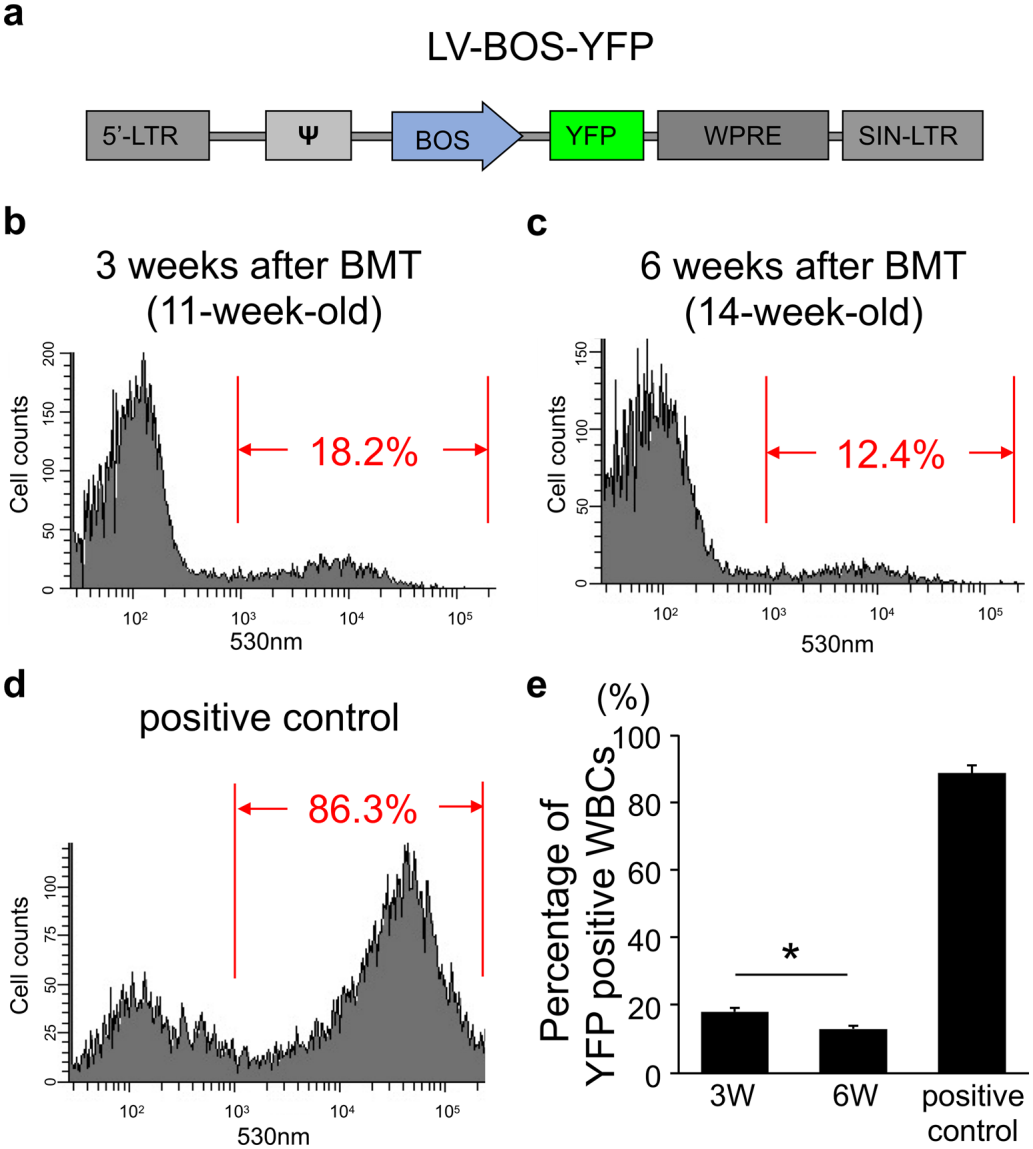
Transduction efficiency of bone marrow cells with LV-BOS-YFP in wild type mice. (**a**) Schematic representation of LV-BOS-YFP. (**b–d**) Representative flow cytometric analysis of peripheral white blood cells with YFP signals after gene transduction by LV-BOS-YFP in wild type mice at 3 weeks [(**b**), 11-week-old] and 6 weeks [(**c**), 14-week-old] after bone marrow transplantation. (**d**) Representative flow cytometric analysis of peripheral white blood cells in recipient mice after bone marrow transplantation from GFP transgenic mice as a positive control. (**e**) The percentage of YFP positive WBCs in total peripheral WBCs of 3 weeks and 6 weeks after bone marrow transplantation groups and positive control group (n = 3, each group). Red letters indicate the YFP-positive population in (**b–d**). Error bars represent the mean + SD. *p < 0.05. *LTR* long terminal repeat, ϕ packaging signal, *WPRE* woodchuck hepatitis virus posttranscriptional regulatory element, *SIN* self-inactivating, *WBCs* white blood cells.

To evaluate cell-based gene transduction efficiency in BMDCs after infection with a lentiviral vector (LV) and BMT, we first constructed a yellow fluorescence protein (YFP) expression vector driven by elongation factor (EF)-1 (BOS) promotor (Fig. 1a, LV-BOS-YFP). After applying the LV-BOS-YFP vector to total BM cells, the cells were used for BMT into wild type adult mice. Three and 6 weeks later, flow cytometric analysis of peripheral blood from the mice was performed. We observed 18.2% and 12.4% YFP positive-cells in a representative mouse white blood cells (WBCs) at 3 and 6 weeks after BMT, respectively (Fig. 1b,c), while 86–91% of WBCs were a GFP-positive population from a representative positive control mouse after BMT from GFP mouse (Fig. 1d,e). The population of YFP-positive cells was significantly decreased from 3 (18.2 ± 1.1%) to 6 weeks (13.2 ± 0.9%) after BMT (Fig. 1e).

### Gene transduction efficiency in BM cells after lentiviral infection and BMT of SOD1(G93A) mice

**Figure 2:**
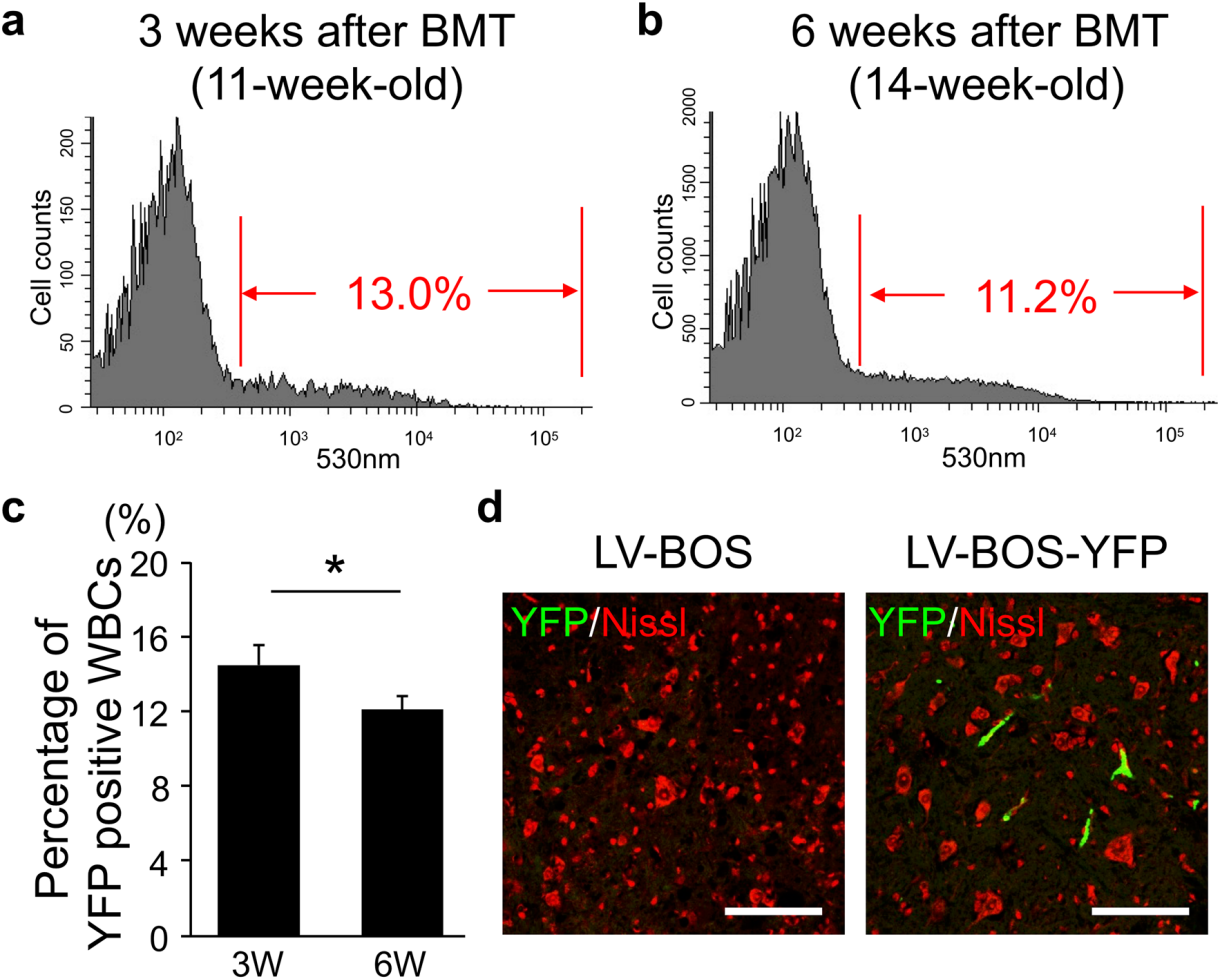
Transduction efficiency of bone marrow cells with LV-BOS-YFP in SOD1(G93A) mice. (**a**,**b**) Representative flow cytometric analysis of LV-BOS-YFP-mediated transduction in SOD1(G93A) mice at 3 weeks [(**a**), 11-week-old] and 6 weeks [(**b**), 14-week-old] after bone marrow transplantation. Red letters indicate the YFP-positive population in (**a**) and (**b**). (**c**) The percentage of YFP positive WBCs in total peripheral WBCs of 3 weeks and 6 weeks after bone marrow transplantation (n = 5, each group). (**d**) YFP signals (green) and Nissl stain (red) in ventral horn sections of the spinal cord at the lumbar level from SOD1(G93A) mice at 6 weeks after bone marrow transplantation with LV-BOS-YFP gene transduction. The left panel shows LV-BOS empty vector group and the right panel shows the LV-BOS-YFP vector group. Scale bar = 100 μm. Error bars represent the mean + SD. *p < 0.05. *WBCs* white blood cells.

Next, the cells were used for BMT into 8-week-old SOD1(G93A) mice after applying the LV-BOS-YFP vector to total BM cells (Fig. 2). We observed 13.0% and 11.2% YFP-positive cells among mouse peripheral WBCs at 3 and 6 weeks after BMT (Fig. 2a,b). The population of YFP-positive cells was significantly decreased from 3 (14.4 ± 1.5%) to 6 weeks (12.1 ± 1.1%) after BMT in ALS mice (Fig. 2c). And, we observed spinal cord tissues to confirm whether these cells had migrated into nervous system tissues. Spinal cords were isolated from SOD1(G93A) mice at 6 weeks after BMT. YFP fluorescent signals were observed beside neurons marked with Nissl stain in sections of the spinal cords in the LV-BOS-YFP group, but we did not detect YFP-positive signals in the SOD1(G93A) mice after BMT transduced with empty vector (LV-BOS) as control group (Fig. 2d). These results indicated that YFP-positive BMDCs had migrated and accumulated in the pathological lesion of the spinal cord under the ALS condition. This suggests the potential of the therapeutic strategy for ALS by cell-based gene delivery to the spinal cord using BMDCs.

**Figure 3:**
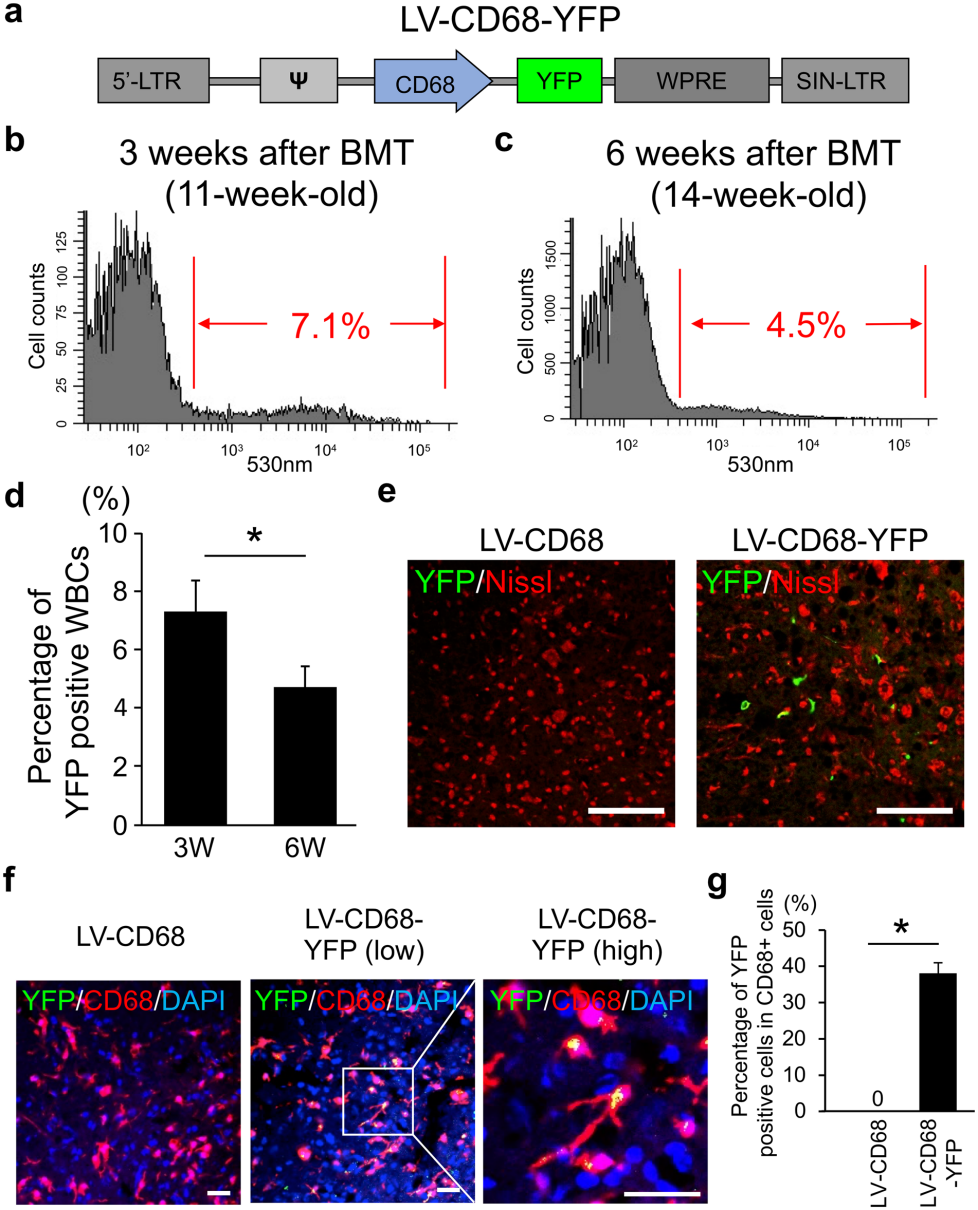
Transduction efficiency of bone marrow cells with LV-CD68-YFP in SOD1(G93A) mice. (**a**) Schematic representation of LV-CD68-YFP. (**b**,**c**) Representative flow cytometric analysis of peripheral white blood cells with YFP signals after gene transduction by LV-CD68-YFP in wild type mice at 3 weeks [(**b**), 11-week-old] and 6 weeks [(**c**), 14-week-old] after bone marrow transplantation. Red letters indicate the YFP-positive population in (**b**) and (**c**). (**d**) The percentage of YFP positive WBCs in total peripheral WBCs of 3 weeks and 6 weeks after bone marrow transplantation (n = 4, each group). (**e**) YFP signals (green) and Nissl stain (red) in ventral horn sections of the spinal cord at the lumbar level from SOD1(G93A) mice after bone marrow transplantation with LV-CD68-YFP gene transduction. The left panel shows in the LV-CD68 empty vector group and the right panel shows in the LV-CD68-YFP vector group. Scale bar = 100 µm. **(f)** Immunohistochemical analysis of CD68 (red) with YFP (green) and DAPI (blue) signals in ventral horn sections of the spinal cord at the lumbar level of SOD1(G93A) mice at 10 weeks (18-week-old) after bone marrow transplantation with gene transduction by LV-CD68-YFP. The left panel shows a low magnification image of the spinal cord in the LV-CD68 empty vector group. The middle panel shows a low magnification image of the spinal cord and the right panel shows a high magnification image of the spinal cord in the LV-CD68-YFP vector group. Scale bar = 50 µm. **(g)** The percentage of YFP positive cells in CD68 positive cells in the spinal cord of LV-CD68 and LV-CD68-YFP groups at 6 weeks after bone marrow transplantation (n = 4, each group). Error bars represent the mean + SD. *p < 0.05. *WBCs* white blood cells.

Furthermore, to develop more specific gene delivery to the spinal cord in ALS mice, the CD68 promoter was used to construct an LV (Fig. 3). Whole BM cells were prepared from wild type mice and infected with the LV expressing YFP protein driven by the CD68 promoter (Fig. 3a, LV-CD68-YFP). These cells were then transplanted into SOD1(G93A) mice. Populations of 7.1% and 4.5% showed YFP gene transduction among peripheral WBCs of SOD1(G93A) mice at 3 and 6 weeks after BMT (Fig. 3b,c). The population of YFP-positive cells was significantly decreased from 3 (7.3 ± 1.1%) to 6 weeks (4.8 ± 0.7%) after BMT in SOD1(G93A) mice (Fig. 3d). In the histological analysis of the spinal cord at 6 weeks after BMT, YFP-positive cells were observed beside neurons marked with Nissl stain in the LV-CD68-YFP group similarly to the LV-BOS-YFP group, whereas we did not detect YFP-positive signals in the empty vector control group (LV-CD68) (Figs. 2d, 3e). YFP positive population in LV-CD68-YFP group was less than in LV-BOS-YFP group in peripheral blood because CD68 population was a part of WBCs. However, YFP positive cells in LV-BOS-YFP group were similarly observed in LV-CD68-YFP group in spinal cord of SOD1(G93A) mice (see Supplementary Fig. 1). These points seem to show that CD68 promoter is more efficient than BOS promoter for gene transduction based on BMDCs in ALS. Additionally, CD68 immunostaining was performed on spinal cord sections of ALS mice at 10 weeks after BMT to confirm that YFP was expressed in CD68-positive cells (Fig. 3f). In the LV-CD68-YFP group, YFP signals were merged with 38.1 ± 2.9% of CD68-positive cells, whereas no YFP signals were observed in the LV-CD68 group (Fig. 3f,g). These results suggested that the LV expressing a therapeutic gene driven by the CD68 promoter may be a powerful tool as a therapeutic strategy for ALS by cell-based gene delivery to the spinal cord using BMDCs.

### Expression level of the transduced gene gradually increases during disease progression in SOD1(G93A) mice

**Figure 4:**
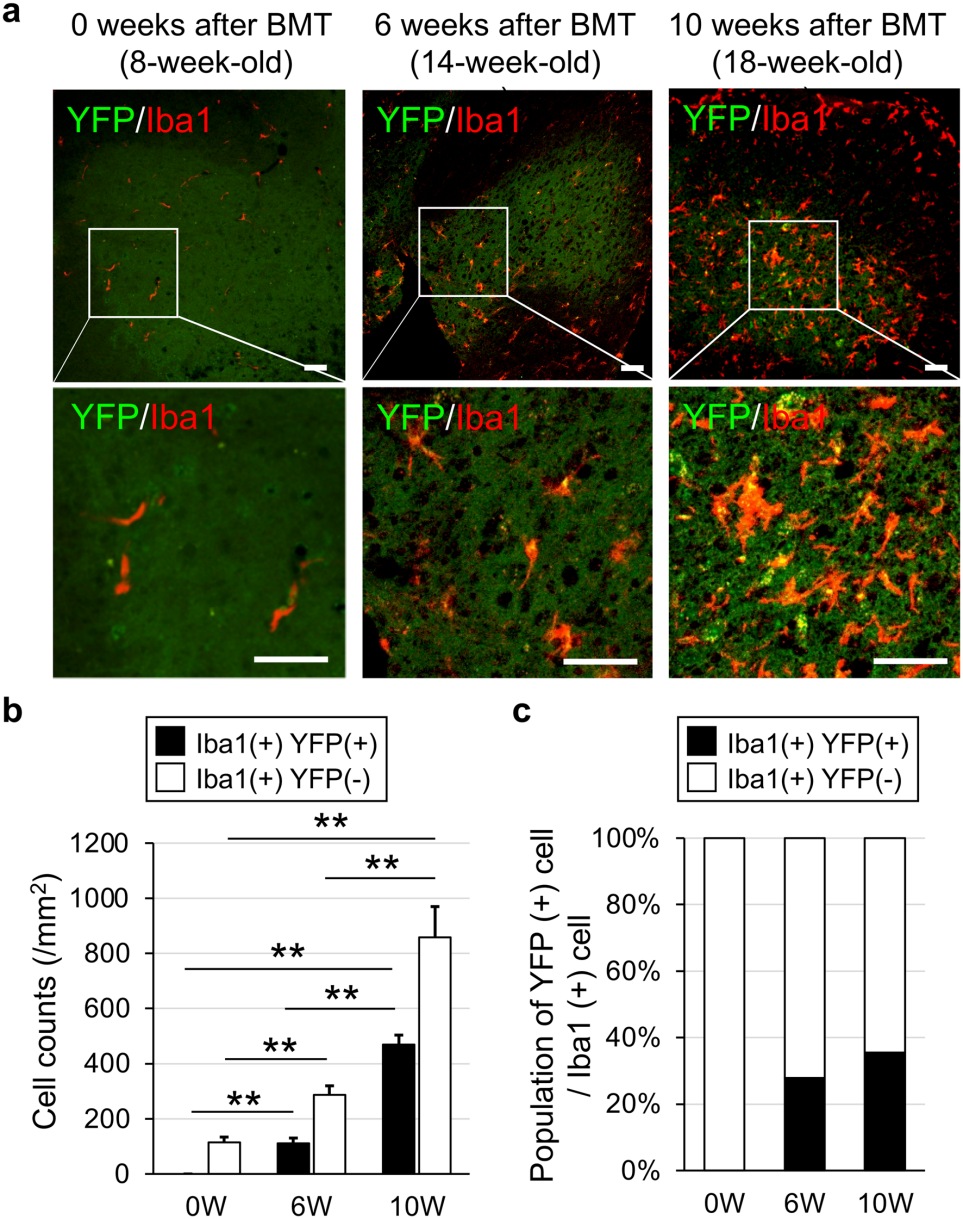
Accumulation of microglia expressing YFP and quantitative analysis of YFP signals in the spinal cord of SOD1(G93A) mice after transplantation. (**a**) YFP (green) and Iba1 (red) signals in ventral horn sections of the spinal cord at the lumbar level in SOD1(G93A) mice at 0 weeks (left panels, 8-week-old), 6 weeks (middle panels, 14-week-old) and 10 weeks (right panels, 18-week-old) after bone marrow transplantation with gene transduction by LV-CD68-YFP. Upper panels show low magnification images of spinal cords and lower panels show a high magnification image of the square in the upper panel of the same column. Scale bar = 50 µm. (**b**) Number of Iba1 and YFP positive cells (Iba1 + YFP +) and Iba1 positive and YFP negative cells (Iba1 + YFP −) in microglia of the spinal cord of SOD1(G93A) mice at 0, 6, and 10 weeks after bone marrow transplantation with gene transduction by LV-CD68-YFP (n = 5, each group). (**c**) Population of YFP positive cells in Iba1 positive cells in microglia of the spinal cord of SOD1(G93A) mice at 0, 6, and 10 weeks after bone marrow transplantation with gene transduction by LV-CD68-YFP (n = 5, each group). Error bars represent the mean + SD. **p < 0.01.

To investigate how the expression level of a delivered gene changed during disease progression of ALS, histological analysis was performed on spinal cords of SOD1(G93A) mice at 0 week as pre-symptomatic state, 6 weeks as mid stage of disease and 10 weeks as end stage of disease, after BMT (Fig. 4). Immunostaining of ionized calcium binding adaptor molecule 1 (Iba1), as a microglial marker, was performed and its correlation with YFP signals was observed at 0, 6 and 10 weeks after BMT (Fig. 4a). Iba1 positive cells were diffusely observed in the spinal cords of ALS mice (Fig. 4a). Most YFP signals were merged with Iba1 staining. Many more Iba1-positive cells in the 10-week group were found compared with the 0-week and 6-week group. Then, to evaluate the origin of Iba1-positive cells of spinal cord in ALS mice, we counted the cell number of Iba1 and YFP double positive cells as BMDCs (Iba1 + YFP +), and that of Iba1 positive and YFP negative cells as resident microglia (Iba1 + YFP −) (Fig. 4b,c). The cell number of both Iba1 + YFP + and Iba1 + YFP − cells gradually increased from 0-week to 6-week group, and from 6-week to 10-week group (Fig. 4b). Additionally, the percentage of Iba + YFP + cells in Iba1 positive population gradually increased as the disease progressed (Fig. 4c). These data suggested that overall YFP expression had increased with the elevation of accumulated Iba1-positive cells in the spinal cord as the disease progressed. Therefore, incorporation of a therapeutic gene into the LV-CD68-YFP vector was considered to deliver more therapeutic gene expression to the spinal cord of ALS mice as the disease progresses.

### GLT1 gene delivery based on BMDCs improved motor function and survival in SOD1(G93A) mice

**Figure 5:**
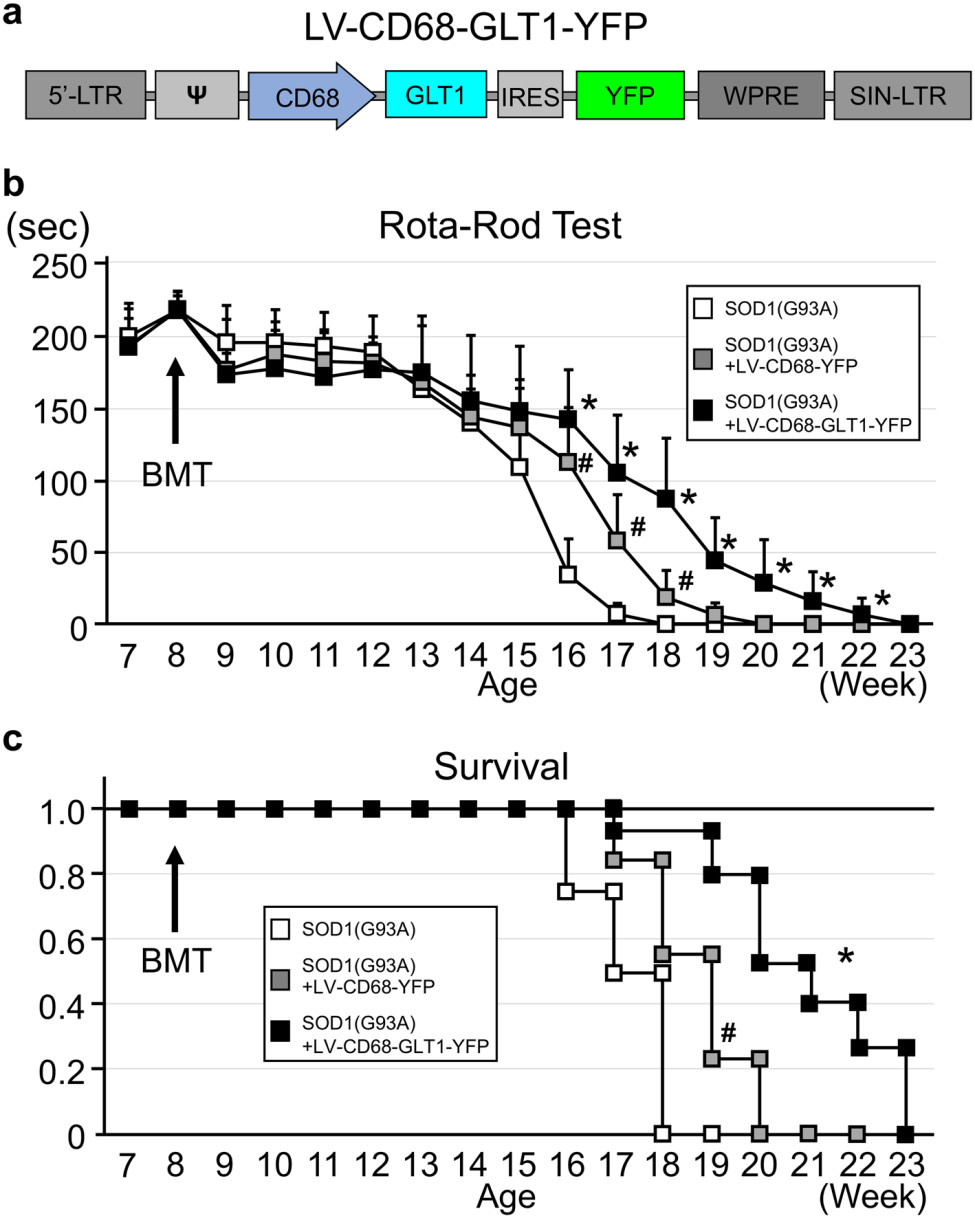
Effect of GLT1 gene delivery with bone marrow transplantation on motor functions and survival of SOD1(G93A) mice. (**a**) Schematic representation of LV-CD68-GLT1-YFP. (**b**) Motor functions were evaluated by the rotarod test once a week in SOD1(G93A) mice (n = 12), SOD1(G93A) mice with [SOD1(G93A) + LV-CD68-GLT1-YFP, n = 15] or without GLT1 gene expression vector treatment [SOD1(G93A) + LV-CD68-YFP, n = 13]. Error bars represent the mean + S.D. *p < 0.05 between SOD1(G93A) + LV-CD68-GLT1-YFP and other two groups, and #p < 0.05 between SOD1(G93A) and SOD1(G93A) + LV-CD68-YFP groups at each time point. (**c**) Kaplan–Meier survival curves in accordance with the survival rate at each week for SOD1(G93A) mice (n = 12), SOD1(G93A) mice with [SOD1(G93A) + LV-CD68-GLT1-YFP, n = 15] or without GLT1 gene expression vector treatment [SOD1(G93A) + LV-CD68-YFP, n = 13]. Y-axis shows the survival rate. *p < 0.05 between SOD1(G93A) + LV-CD68-YFP and SOD1(G93A) + LV-CD68-GLT1-YFP groups and #p < 0.05 between SOD1(G93A) and SOD1(G93A) + LV-CD68-YFP groups with the log-rank test. *IRES* internal ribosome entry site.

For treatment of ALS model mice, GLT1 was inserted into the LV-CD68-YFP vector (LV-CD68-GLT1-YFP, Fig. 5a). To investigate whether GLT1 suppressed disease development, BM cells from wild type mice were infected with LV-CD68-GLT1-YFP and BMT using these cells was performed in 8-week-old SOD1(G93A) mice. After BMT, motor functions and survival were evaluated by the rotarod test and the physiological condition was monitored each week until physiological death and compared with SOD1(G93A) mice transplanted with non-infected BM from SOD1(G93A) mice or LV-CD68-YFP-infected BM from wild type mice (Fig. 5b,c). SOD1(G93A) mice in the LV-CD68-GLT1-YFP group showed significant improvement of motor behaviors compared with those in the SOD1(G93A) and in the LV-CD68-YFP control groups at 16–22 week (Fig. 5b). And LV-CD68-YFP control group showed significant improvement of motor behaviors compared with those in the SOD1(G93A) group at 16–18 week (Fig. 5b). In addition, Kaplan–Meier curves showed that the survival rate was significantly prolonged in the LV-CD68-GLT1-YFP group compared with the LV-CD68-YFP group, and in the LV-CD68-YFP group compared with SOD1(G93A) group, similarly to the trend of motor functions (Fig. 5c). These results indicate that BMDCs expressing GLT1 is most effective, however BMT alone from wild type mice is also effective.

### GLT1 protein and gene expression and glutamate contents in SOD1(G93A) mice after gene therapy

**Figure 6:**
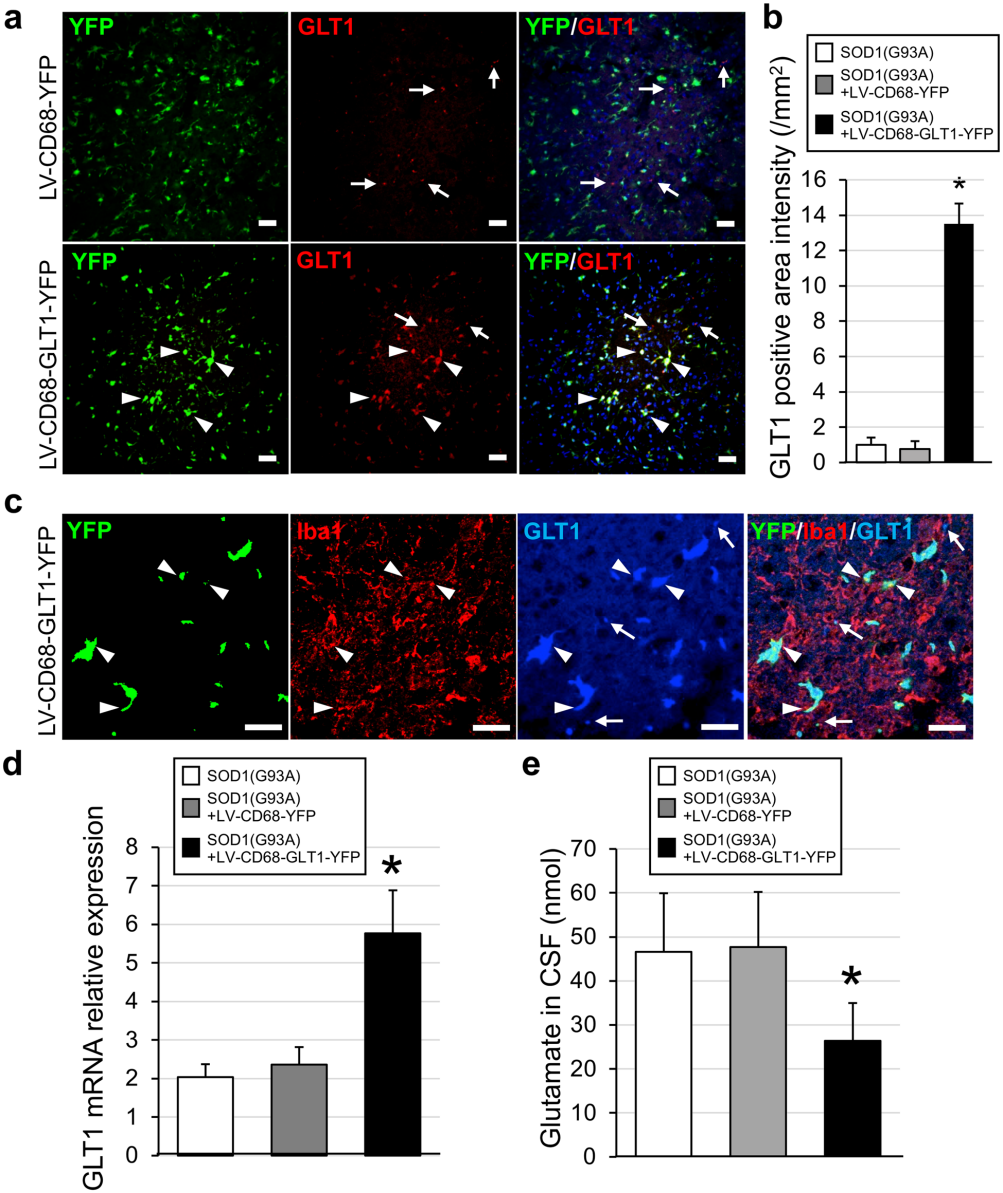
Immunohistochemistry of GLT1 with Iba1, mRNA expression of GLT1 and glutamate contents in the of SOD1(G93A) mice after treatments. (**a**) Immunohistochemical analysis of GLT1 (red) with YFP (green) signals at ventral horn in the spinal cord of SOD1(G93A) mice at 10 weeks after bone marrow transplantation with gene transduction by LV-CD68-YFP (upper row panels) or LV-CD68-GLT1-YFP (lower row panels). Left panels show YFP signals, middle panels show GLT1 staining, and right panels show merged images of left and middle panels in the same row. Arrowheads indicate YFP and GLT1 double-positive cells. Arrows indicate GLT1 single-positive cells. Scale bar = 50 µm. (**b**) GLT1-positive area intensity in SOD1(G93A) mice (n = 6) and SOD1(G93A) mice with the empty vector [SOD1(G93A) + LV-CD68-YFP, n = 6] or treatment vector [SOD1(G93A) + LV-CD68-GLT1-YFP, n = 6]. Error bars represent the mean + SD. *p < 0.05 between the treatment group and others. (**c**) Immunohistochemical analysis of Iba1 (red) and GLT1 (blue) with YFP (green) signals at ventral horn in the spinal cord of SOD1(G93A) mice at 10 weeks after bone marrow transplantation (18-week-old) with gene transduction by LV-CD68-GLT1-YFP. The most left panel shows YFP signals (green), the second, third and fourth panel from the left side show Iba1 (red), GLT1 (blue) staining and the merged images of three left side panels. Arrowheads indicate YFP, Iba1 and GLT1 triple-positive cells. Arrows indicate GLT1 positive cells without YFP signals. Scale bar = 50 µm. (**d**) mRNA expression level of GLT1 in SOD1(G93A) mice (n = 6) and SOD1(G93A) mice with the empty vector [SOD1(G93A) + LV-CD68-YFP, n = 6] or treatment vector [SOD1(G93A) + LV-CD68-GLT1-YFP n = 6]. Data were normalized to GAPDH mRNA expression. (**e**) Glutamate concentration in cerebrospinal fluid in SOD1(G93A) mice, SOD1(G93A) mice with the empty vector [SOD1(G93A) + LV-CD68-YFP] or treatment vector [SOD1(G93A) + LV-CD68-GLT1-YFP] (n = 4 in each group). Error bars represent the mean + SD. *p < 0.05 between the treatment group and others. *CSF* cerebrospinal fluid.

To analyze the mechanism underlying the beneficial effects of LV-CD68-GLT1-YFP in SOD1(G93A) mice, immunohistochemistry of GLT1 was performed on the spinal cords of LV-CD68-YFP and LV-CD68-GLT1-YFP groups, and the immunostaining and YFP signals were observed together (Fig. 6a). A large number of YFP-positive areas were diffusely observed throughout the spinal cords of both LV-CD68-YFP and LV-CD68-GLT1-YFP groups. However, GLT1-positive cells in the LV-CD68-GLT1-YFP group were clearly and abundantly present, which overlapped with YFP signals (Fig. 6a, arrowheads), whereas GLT1-positive staining was only slightly detected in the LV-CD68-YFP group. Additionally, because few GLT1-positive areas in the LV-CD68-YFP group did not overlap with YFP (Fig. 6a, arrows), these findings suggested that most of the GLT1-positive areas in the LV-CD68-GLT1-YFP group had a BM origin, whereas those areas in the LV-CD68-YFP group had an endogenous origin rather than BM. Quantitative analysis revealed that the intensity of the GLT1-positive area was approximately three times larger in the LV-CD68-GLT1-YFP group than in SOD1(G93A) disease control and LV-CD68-YFP groups (Fig. 6b). To clarify the relation of Iba1 positive cells and bone marrow-derived YFP positive cells with GLT1 expression, double staining of Iba1 and GLT1 was performed in LV-CD68-GLT1-YFP treatment group (Fig. 6c). Many Iba1 positive cells were diffusely observed in over the ventral horn of the spinal cord and some population of them were partly merged with YFP and GLT1 (Fig. 6c, arrowheads). And many YFP negative and Iba1 positive cells were also observed. And small number of YFP negative Iba1 negative cells expressed GLT1 (Fig. 6c, arrows). Additionally, quantitative analysis of GLT1 mRNA was performed on the spinal cords of SOD1(G93A), LV-CD68-YFP, and LV-CD68-GLT1-YFP groups. Similarly, GLT1 gene expression was particularly increased in the LV-CD68-GLT1-YFP group compared with the other groups (Fig. 6d). Furthermore, to confirm the protective action of GLT1, we measured glutamate contents in cerebrospinal fluid (CSF) of SOD1(G93A), LV-CD68-YFP, and LV-CD68-GLT1-YFP groups (Fig. 6e). The concentration of glutamate in CSF was significantly decreased in the LV-CD68-GLT1-YFP group compared with the other two groups (Fig. 6e). These data suggested that more exogenous GLT1 was expressed in the spinal cords of the LV-CD68-GLT1-YFP group, which indicated that cells expressing the therapeutic gene was efficiently delivered to the pathological lesion in the spinal cord of SOD1(G93A) mice.

### Histological analysis of motor neuron survival, astrogliosis, muscle atrophy and nerve degeneration in SOD1(G93A) mice after gene therapy

**Figure 7:**
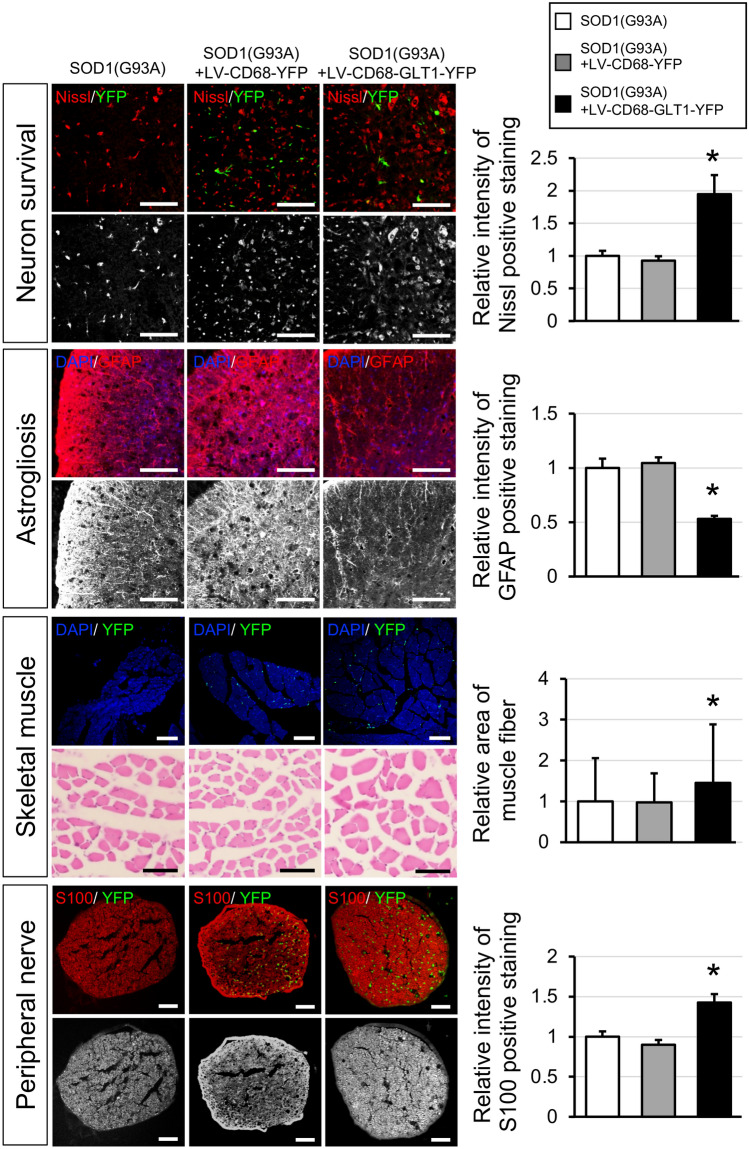
Histological analysis of motor neuron survival and astrogliosis in spinal cord, degeneration of muscle and peripheral nerve in SOD1(G93A) mice after treatments. The first and second row from the top: Nissl stain (red) with YFP (green) signals of the spinal cords in the SOD1(G93A), SOD1(G93A) + LV-CD68-YFP and SOD1(G93A) + LV-CD68-GLT1-YFP mice at 18–20-week-old. Upper row shows the color images and lower row shows black and white images of red color (Nissl staining) isolated from the corresponding upper row. The third and fourth row from the top: GFAP immunohistochemistry (red) with YFP (green) signals of the spinal cords in the same three group. Upper row shows the color images and lower row shows black and white images of red color (GFAP staining) isolated from the corresponding upper row. The third and fourth row from the bottom: upper row shows YFP (green) signals with a nuclear stain (DAPI, blue) of the spinal cords in the same three group. Lower row shows Hematoxylin–eosin stain of anterior tibial muscle in the same three group. The first and second row from the bottom: S100 immunohistochemistry (red) with YFP (green) signals of the spinal cords in the same three group. Upper row shows the color images and lower row shows black and white images of red color (S100 staining) isolated from the corresponding upper row. Scale bar = 100 µm. The bar graph shows relative intensity of Nissl, GFAP and S100 staining as seen in the same three group (n = 5 in each group). The intensity of Nissl, GFAP and S100 staining was measured in the black and white image using the Image J software and the ratio was calculated against the intensity of that in SOD1(G93A) group. The bar graph shows relative area of muscle fiber in the same three groups. The average areas of muscle fibers were compared among the three groups (n = 5 in each group). Error bars represent the mean + SD. **p < 0.01 between the treatment group and others.

To confirm the protective effects of GLT1 expression by BMDCs, motor neuron survival, astrogliosis, muscle atrophy and nerve degeneration were analyzed in the LV-CD68-GLT1-YFP group and compared with the SOD1(G93A) and the LV-CD68-YFP groups (Fig. 7). In spinal cord sections, Nissl stain for survival neurons was performed at ventral horn in the SOD1(G93A), in the LV-CD68-YFP and in the LV-CD68-GLT1-YFP groups (Fig. 7, Neuron survival). Motor neurons were preserved in LV-CD68-GLT1-YFP group more than other two groups, and intensity of Nissl stain was approximately twice higher than the other groups (Fig. 7, most upper two rows and their right-side bar graph). For the evaluation of astrogliosis, GFAP immunostainings and their quantification were performed in same three groups (Fig. 7, Astrogliosis). GFAP staining in the LV-CD68-GLT1-YFP group was the weakest among the three groups, and the staining was suppressed to approximately half that of the other two groups (Fig. 7, third and fourth rows and their right-side bar graph). Next, we evaluated skeletal muscle and peripheral nerve as the peripheral compartment (Fig. 7, Skeletal muscle and Peripheral nerve). In both muscle and nerve, YFP positive signals were observed patchy in the LV-CD68-YFP and in the LV-CD68-GLT1-YFP groups, and they were similar frequent in the two groups (Fig. 7, fifth and seventh rows, and Supplementary Fig. 2). However, muscle fiber area and S100 protein, as a myelin marker, were significantly preserved in the LV-CD68-GLT1-YFP group compared to the other two control groups (Fig. 7, sixth and eighth rows and their right-side bar graphs). In addition, the distribution of muscle fiber area was shifted to right side in the LV-CD68-GLT1-YFP group compared to the other two groups (See Supplementary Fig. 3). These results suggested that GLT1 expression provided by BMDCs protected pathological changes both in the spinal cord and in peripheral nervous system.

### Gene expression of cytokines and neuroprotective microglia markers in SOD1(G93A) mice after gene therapy

**Figure 8:**
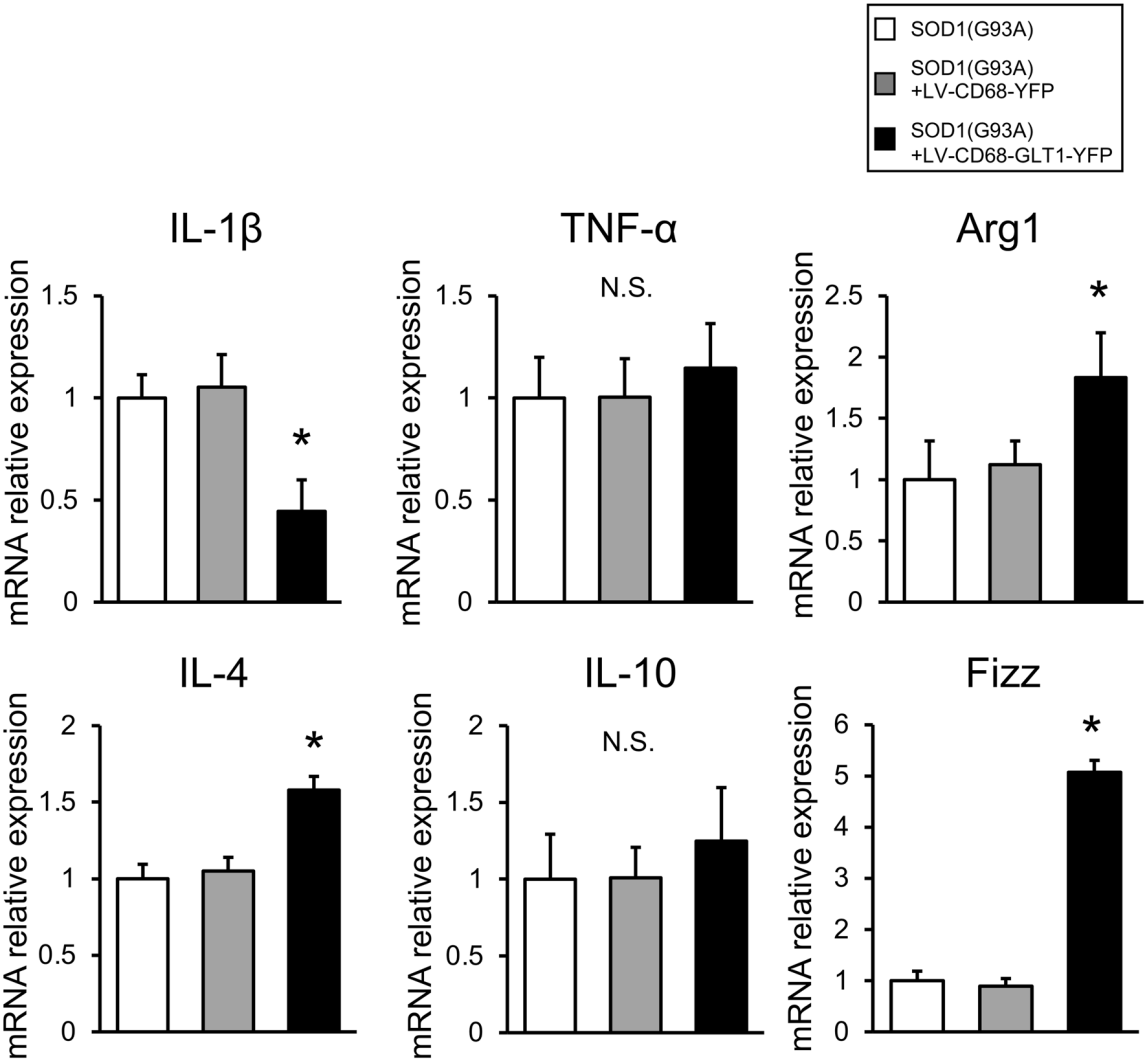
Quantitative gene analysis of the spinal cord of SOD1(G93A) mice after transplantation. mRNA expression of IL-1β, TNF-α, IL-4, IL-10, Arg1, and FIZZ was evaluated in the spinal cord of SOD1(G93A), SOD1(G93A) + LV-CD68-YFP and SOD1(G93A) + LV-CD68-GLT1-YFP groups at 18–20-week-old (n = 5 each group). Data were normalized to GAPDH mRNA expression. Error bars represent the mean + SD. *p < 0.05 between the treatment group and others.

To clarify the mechanism of the therapeutic effects by GLT1 gene delivery and expression, we performed quantitative PCR analyses of inflammatory and anti-inflammatory cytokines, and neuroprotective microglia markers in the spinal cord tissues of treated SOD1(G93A) mice (Fig. 8). Expression of IL-1β as an inflammatory cytokine was significantly suppressed in the LV-CD68-GLT1-YFP group, and that of IL-4 as an anti-inflammatory cytokine was significantly increased in the LV-CD68-GLT1-YFP group compared with the other two groups (Fig. 8). Additionally, expression of arginase (Arg) 1 and FIZZ as neuroprotective microglia markers was significantly increased in the LV-CD68-GLT1-YFP group (Fig. 8). However, expression of TNF-α as an inflammatory cytokine and that of IL-10 as an anti-inflammatory cytokine were not different in the LV-CD68-GLT1-YFP group compared with the other two groups (Fig. 8). These results suggested that expression of GLT1 in the pathological lesion induced a beneficial environment for neurons in the spinal cord.

## Discussion

Apoptosis, inflammation, oxidative stress, and excitotoxicity have been implicated in the pathogenesis of ALS^28–30^. Numerous studies have reported that novel treatments including stem cell therapy, administration of growth factors, and gene therapy might prolong survival and delay the progression of symptoms^3–8,19^. Stem cells have emerged as an attractive option to treat ALS because they tend to migrate to damaged nerves, which offers a means to deliver therapeutic genes where they are needed^8,31,32^. Gene therapy may facilitate curing ALS if vectors can carry therapeutic genes to salvage dying nerve cells. Our current study combined gene therapy with stem cells, which showed that BMDCs expressing GLT1 by the LV in transgenic SOD1(G93A) mice resulted in expression of GLT1 at segments of the spinal cord and led to an extension of lifespan and improved motor functions.

The pathogenesis involved in motor neuron death of ALS is complex and neuroinflammation has been accepted as a major contributor to motor neuron degeneration and disease progression^33^. Recently, it was noted that non-neuronal cells, such as immune cells, endothelial cells, and glial cells surrounding neurons, are deeply involved in the pathogenesis of neurodegenerative diseases in addition to the autonomous neuronal cell death caused by accumulated abnormal proteins^34,35^. This theory of “non-cellular autonomic nerve cell death” is also attracting attention in terms of ALS, a neurodegenerative disease^34,35^. Microglia are distributed throughout the brain and spinal cord parenchyma, which account for 10%–20% of the total glial cell population^36^.

We have reported that a large number of microglia accumulate in the spinal cord as the stage progresses in an ALS model animal^19^. Similarly, our results showed that YFP expression increased with the elevation of accumulated Iba1-positive cells in the spinal cord as the disease progressed in this study. Therefore, the migrating YFP-positive cells acted as gene carriers and the number of migrating cells was increased gradually in the spinal cord as the stage progressed, which increased the expression of therapeutic genes.

Microglia are extremely sensitive to physiological changes in their environment and become “activated” following exposure to specific cytokines and growth factors, which indicates infection, trauma, neuronal insult, or inflammation^37^. The levels of several proinflammatory cytokines are altered during ALS, which suggest the presence of inflammation. During chronic neuroinflammation, CNS-infiltrating macrophages express microglial markers and convert to significantly different phenotypes^38–40^. Microglia primarily have two different phenotypes, proinflammatory and neuroprotective, in response to various microenvironmental signals^41^. On the basis of different activation stimuli, microglia polarize to either the proinflammatory or neuroprotective type. During the early stage of motor neuron injury in ALS models, the surveying microglia exhibit the neuroprotective phenotype^42^. However, as the disease progresses, microglia shift to the proinflammatory phenotype and injure motor neurons^43^. In this study, expression of IL-1β by proinflammatory microglia was significantly suppressed in the LV-CD68-GLT1-YFP group. This is in agreement with reports showing that chronic administration of IL-1β results in neurodegeneration^44^, whereas IL-1β depletion attenuates inflammation and prolongs the lifespan of ALS mouse models^45^. Additionally, expression of IL-4, Arg1, and Fizz as the neuroprotective microglial markers^46,47^ was significantly increased in the LV-CD68-GLT1-YFP group, which may have been expressed by the infiltrating BMDCs transformed to Iba1-positive microglia-like cells, or the induced resident neuroprotective microglia in spinal cord with gene therapy. CNS delivery of IL-4 in SOD1(G93A) mice via a lentiviral-mediated gene therapy strategy has resulted in general amelioration of clinical outcomes during the early slowly progressive phase of the disease^48^. Therefore, the migration of BMDCs and expression of GLT1 in the spinal cord are thought to have improved the environment around neurons from inflammatory to non-inflammatory. In addition, as a direct effect of GLT1 expression in BMDCs, glutamate content was significantly reduced in CSF from the treated mice. This reduction could be caused by taking glutamate into BMDCs through GLT1. It has been reported that the loss of GLT1 induce increased extracellular levels of glutamate and cause motor neuron toxicity and muscle paralysis in animal models^49^, and the pharmacological stimulation of GLT1 rescue motor neuron degeneration in SOD1(G93A) mice^50^. Excess glutamate, which has been observed in CSF in ALS patients, has been reported to be toxic to neuron^51^. Therefore, these results support that the treatments have protected neurons from neurotoxicity of glutamate. Furthermore, as astrogliosis, muscle atrophy and peripheral nerve degeneration were significantly suppressed, the infiltrating BMDCs and their expression of GLT1 may have affected astrocyte in spinal cord, muscle cells and Schwann cells in peripheral tissues in ALS mice.

As a represent study of BM cell-based gene therapy, treatment of adrenoleukodystrophy (ALD) has been performed. ALD is a genetic disease linked to X chromosome and caused by the dysfunction of peroxisomal ATP-binding cassette (ABC) half-transporter ALD protein due to the mutation of ABC, subfamily D, member 1 (ABCD1) gene^52^. Cerebral ALD shows demyelination and neurodegeneration^52^. Progression of the disease results in loss of neurological functions and death, which can be stopped only by the transplantation of allogeneic hematopoietic stem cells^52^. Recently, a cell transplantation therapy of the autologous CD34 + cells transduced by an elivaldogene tavalentivec (Lenti-D) LV has been reported^53^. This study indicates that Lenti-D gene therapy safely and effectively halts the progression of ALD and may be offered instead of allogeneic stem cell transplantation in patients with cerebral ALD^53^. This study supports the feasibility of our strategy because it is a clinical application of BM cell-based gene therapy. Recently, a literature reported that bone marrow derived-cells were not accumulated into spinal cord of ALS mice without irradiation with BMT^54^. However, our finding that many BMDCs accumulate in the spinal cord as the disease progresses is very attractive, even with the effects of irradiation. In fact, BMT has been previously performed on ALS patients after total body irradiation^55^. Therefore, it is believed that our strategy seems to be acceptable as a clinical application while further safety should be confirmed.

In conclusion, this study indicates that BMDC-based gene delivery of GLT1 ameliorates motor functions and survival of SOD1(G93A) mice.

## Materials and methods

### Animals

C57BL/6, hSOD1 (G93A) 1Gur/J [SOD1(G93A) mice] and C57BL/6-Tg (UBC-GFP) ^30Scha/J^ (GFP mice) were prepared by purchasing from Jackson Laboratories (Bar Harbor, ME). Water and mouse chow were provided to all animals ad libitum, and they were kept in a 12-h light and dark cycle. Animal experimental protocols have been approved by the Institutional Animal Care and Usage Committee (IACUC) of Shiga University of Medical Science. And all experimental protocol were performed in accordance with the guidelines of the IACUC at Shiga University of Medical Science. This study was also carried out in compliance with the ARRIVE guidelines.

### Lentiviral vectors

pLL3.7 plasmids including the EF-1 promoter with or without YFP as a reporter gene (pLL3.7-BOS and pLL3.7-BOS-YFP, respectively) were obtained from Addgene (Watertown, MA). pLL3.7-CD68 and pLL3.7-CD68-YFP plasmids were constructed by exchanging the EF-1 promoter with the CD68 promoter. The full length GLT1-coding sequence (NM_001077514.3) was isolated from primary astrocytes of the mouse brain at postnatal day 1. Full length mouse GLT1 cDNA was inserted into LL3.7-CD68-YFP (GLT-1 over-expression plasmid: LL3.7-CD68-GLT1-IRES-YFP). To produce LVs, psPAX2 (packaging plasmid), pMD2.G (envelope plasmid encoding the vesiculo-stomatitis virus G-protein), and each LL-3.7-based plasmid (pLL3.7-BOS, pLL3.7-BOS-YFP, pLL3.7-CD68, pLL3.7-CD68-YFP, and pLL3.7-CD68-GLT1-IRES-YFP) were transfected into 293 T cells using calcium phosphate as described previously^56^. The culture complete media were changed after 6 h of incubation at 37 °C. LVs were collected from the supernatant of culture media after 48 h of incubation and was passed through a 0.45 µm pore size cellulose acetate filter. LVs were concentrated using a Lenti-X Concentrator (Takara Bio Inc., Otsu, Japan)^56^. After determination of infectious units (IFU) by 293 T cells, LVs were stored at a concentration of 1.8 × 10^8^ IFU/ml at − 80 °C. Finally, we produced LV-BOS, LV-BOS-YFP, LV-CD68, LV-CD68-YFP, and LV-CD68-GLT1-YFP (GLT1 was expressed with YFP).

### Isolation of bone marrow cells and infection of lentiviral vectors

Whole BM cells were isolated from wild type C57BL/6 mice as described previously^19^. Before BMT into wild type or SOD1(G93A) mice, 1 × 10^6^ BM cells per mouse were infected with LVs at a concentration of 10 MOI (100 vp/cell) in 1 ml Span Serum-free expansion medium (STEMCELL Technologies, Vancouver, Canada) with stem cell factor (100 ng/ml; R&D Systems, Minneapolis, MN), Flt3 (100 ng/ml; R&D Systems), IL-3 (100 ng/ml; R&D Systems), and IL-6 (200 ng/ml; R&D Systems). After 12 h of incubation at 37 °C, the lentivirus-infected BM cells were used for BMT as gene therapy for SOD1(G93A) mice. Before BMT, transduction efficiency of all LVs to BM cells were confirmed.

### Bone marrow transplantation and gene therapy of SOD1(G93A) mice

For BMT, 8-week-old female SOD1(G93A) and wild type female C57BL/6 mice were irradiated (9 Gy) and then injected from tail vein with 1 × 10^6^ whole BM cells after lentiviral infection. After BMT, these mice were used to evaluate gene transduction efficiency and gene therapy.

### Flow cytometric analysis

Transduction efficiency of the transferred gene was evaluated by flow cytometric analysis in WBCs from wild type and SOD1(G93A) mice at 3 and 6 weeks after BMT because it takes approximately three to four weeks for the stable engraftment of bone marrow cells after transplantation. WBCs in 200 µl blood were collected from each mouse by cutting the tip of tail, and centrifuged at 100×*g* for 5 min at 4 °C. After the pellet of blood cells was resuspended with 1 ml lysis buffer (154 mM NH_4_Cl, 2 mM NaHCO_3_ and 0.1 mM EDTA_2_Na), that was centrifuged at 100×*g* for 5 min at 4 °C. Then, 1 ml PBS (−) was added to the cell pellet, followed by centrifugation at 100×*g* for 5 min at 4 °C again. After 500 µl PBS (−) was added to the cell pellet, for the cells were analyzed by flow cytometry. Emission at 530 was induced by excitation of a 488 nm laser. The gate of the fluorescence threshold was determined using WBCs from C57BL/6 mice after BMT from GFP or wild type mice as positive and negative controls, respectively. A FACS Canto II with FACS DIVA software (BD Biosciences) was used for data collection and analysis.

### Quantitative RT-PCR analysis

The mice were transcardially perfused with PBS and the spinal cord tissues at the lumbar level were collected under deep anesthesia by intraperitoneal injection of medetomidine (0.3 mg/kg), midazolam (4 mg/kg) and butorphanol (5 mg/kg). Tissues were immediately frozen in liquid nitrogen after isolation. A RNeasy mini kit (Qiagen, Valencia, CA) was used for total RNA extraction from isolated tissues after DNase I (RNase-free DNase set, Qiagen) digestion. Reverse transcription was performed using 100 ng of total RNA and Prime Script perfect Real time (Takara Bio Inc.). RT-PCR was performed by using the following primers: forward 5′-CTTGGCTTGCTTCGGAACTC-3′ and primer 5′-GGAGAAGGCGTTTGCTTAGTTC-3′ for Arg1, forward 5′-GCCAGGTCCTGGAACCTTTC-3′ and reverse 5′-GGAGCAGGGAGATGCAGATGAG-3′ for Fizz, forward 5′-ATGACCACAGTCCATGCCATC-3′ and reverse 5′-GAGCTTCCCGTTCAGCTCTG-3′ for mouse GAPDH, forward 5′-TAACTCTGGCGGCCAATGGAAAGT-3′ and reverse 5′-TTATTTTTCACGTTTCCAA-3′ for GLT1, forward 5′-CAACCAACAAGTTGATATTCTCCATG-3′ and reverse 5′-GATCCACACTCTCCAGCTGCA-3′ for IL-1β, forward 5′-TCAACCCCCAGCTAGTTGTC-3′ and reverse 5′-TGTTCTTCGTTGCTGTGAGG-3′ for IL-4, forward 5′-CCAAGCCTTATCGGAAATGA-3′ and reverse 5′-TTTTCACAGGGGAGAAATCG-3′ for IL-10, forward 5′-CACGTCGTAGCAAACCACCAAGTGG-3′ and reverse 5′-GATAGCAAATCGGCTGACGGTGTGG-3′ for Tnf-α. To quantify each gene, real-time PCR was performed according to the SYBR Green protocol with a LightCycler 480 (Roche Diagnostics, Manheim, Germany). The PCR condition was one cycle of 95 °C for 3 min, followed by 45 cycles of denaturation at 95 °C for 30 s, annealing at 60 °C for 30 s and extension at 72 °C for 30 s. The emitted fluorescence was measured three times during the annealing extension phase. Amplification plots were analyzed by LightCycler 480 software, version 1.5 (Roche Diagnostics)^55^. Genomic DNA contamination was eliminated by the DNase digestion. Each gene expression was analyzed using the 2 − ΔΔCt method and calculated the relative expression with normalization by GAPDH as the control.

### Behavior test

Rotarod tests (Ugo Basile, Comerio-Varese, Italy) were conducted once a week from 1 week before the beginning of treatment to physiological death (when the rotarod test result was 0 s, the mice were considered as physiologically dead). Rotarod tests were performed at a rotation speed of 5 rpm/min to a maximum of 50 rpm/min for 5 min (acceleration was 9 rpm/min^2^) as previously described^19^. The averages of three medians in five trials for each mouse with an interval of > 3 min were calculated and used to evaluate motor functions^19^. SOD1(G93A) mice were grouped in a random manner. Rota-rod test was performed in a non-blinded manner. Mice were adjusted age and sex, and the tests were performed by the same enforcer. The number of living mice was counted in accordance with the definition of physiological death until all mice were recognized as such for the analysis of Kaplan–Meier survival curve^19^. And there was no censoring of mice due to treatment-related death.

### Histological analysis

For histological analysis, SOD1(G93A) mice after treatment were transcardialy perfused with PBS and fixed with 4% paraformaldehyde. Then, the spinal cord at the lumbar level, anterior tibialis muscle and sciatic nerves were isolated and their sections were prepared by embedding with OCT compound (Sakura Finetek Japan, Tokyo, Japan) and cutting. For immunostaining, the sections were incubated with a primary antibody (anti-rabbit Iba1 [1:200, Wako, Osaka, Japan], anti-mouse CD68 [1:100, Santa Cruz Biotechnology, Carlsbad, CA], anti-rabbit GFAP [1:200, Cell Signaling Technology, MA, USA], anti-rabbit S100 [1:200, abcam, Cambridge, UK] or anti-rabbit GLT1 [1:200, Cell Signaling, Danvers, MA] at 4℃ overnight. Then, the sections were incubated with a secondary antibody (anti-rabbit Alexa 555, anti-mouse Alexa 555 or anti-rabbit Alexa 633 [Molecular Probes, Eugene, OR]) at room temperature for 4 h and mounted with the Vectashield mounting medium with or without DNA staining using 4′,6-diamidino-2-phenylindole (DAPI; Vector Laboratories, Burlingame, CA). Some sections of spinal cords were performed Nissl stain with NeuroTrace 530/615 (Thermo Fisher Scientific) for the analysis of degeneration of motor neuron^57^. These sections were observed under a confocal laser microscope (C1si; Nikon, Tokyo, Japan) with EZC1 3.90 software (Nikon). For intensity analysis of Nissl stain, YFP signals, immunostainings of GFAP, GLT1 or S100, ImageJ software version 1.52a was used (National Institutes of Health, Bethesda, MD). For quantitative analysis, Nissl-positive staining, YFP signals and GFAP-GLT1- or S100-positive immunostaining were converted to the black white image after splitting to single color, and the intensity was measured in over ten scenes of each mouse by ImageJ software version 1.51 (National Institutes of Health, Bethesda, MD)^57^. To analyze the muscle degeneration, the sections were stained with hematoxylin–eosin or mounted with the Vectashield mounting medium with DAPI (Vector Laboratories)^57^. The muscle fibers’ area was measured in over ten scenes of each mouse by ImageJ software version 1.51 (National Institutes of Health).

### Glutamate assay

For glutamate assay, the cerebrospinal fluid was collected from the cisterna magna of 18-week-old SOD1(G93A) mice after transplantation. The concentration of the glutamate levels in the cerebrospinal fluid were measured by Glutamate Assay Kit according to the manufacturer’s protocol (ab83389, abcam).

### Statistical analysis

Statistical analysis was performed using SPSS 25.0 software (IBM Corp., Armonk, NY). All data are shown as the mean ± standard deviation (S.D.). One-way ANOVA and Tukey’s test was used for analysis of statistical significance for multiple datasets. The log-rank test was performed for statistical analysis of the Kaplan–Meier curve for a univariate survival. The analysis was performed with GLT1 treatment as an independent variable and the survival as a dependent variable. Data were considered significantly different at p < 0.05.

## Supplementary Information

Supplementary Figures.

## Data Availability

All the data for this study will be available upon reasonable request to the corresponding author.
