# Supplementary material for: GLT1 gene delivery based on bone marrow-derived cells ameliorates motor function and survival in a mouse model of ALS
Source: Sci Rep. 2021 Jun 17;11:12803. doi: 10.1038/s41598-021-92285-x (PMC8211665; doi:10.1038/s41598-021-92285-x)
Supplement: Supplementary file 1 — Supplementary Figures. [file 41598_2021_92285_MOESM1_ESM.pdf]

GLT1 gene delivery based on bone marrow-derived cells ameliorates motor function and survival in a mouse model of ALS

[1]Natsuko Ohashi, [1]Tomoya Terashima, [1]Miwako Katagi, [1]Yuki Nakae, [2]Junko Okano, [2]Yoshihisa Suzuki and [1]Hideto Kojima

[1]Department of Stem Cell Biology and Regenerative Medicine, [2]Department of Plastic and Reconstructive Surgery, Shiga University of Medical Science, Shiga, JAPAN

Address correspondence to: Tomoya Terashima, Department of Stem Cell Biology and Regenerative Medicine, Shiga University of Medical Science, Seta Tsukinowa-cho, Otsu, Shiga 520-2192, JAPAN

E-mail: tom@belle.shiga-med.ac.jp
Tel: +81-77-548-2207, Fax: +81-77-548-2642

Supplementary Figure 1

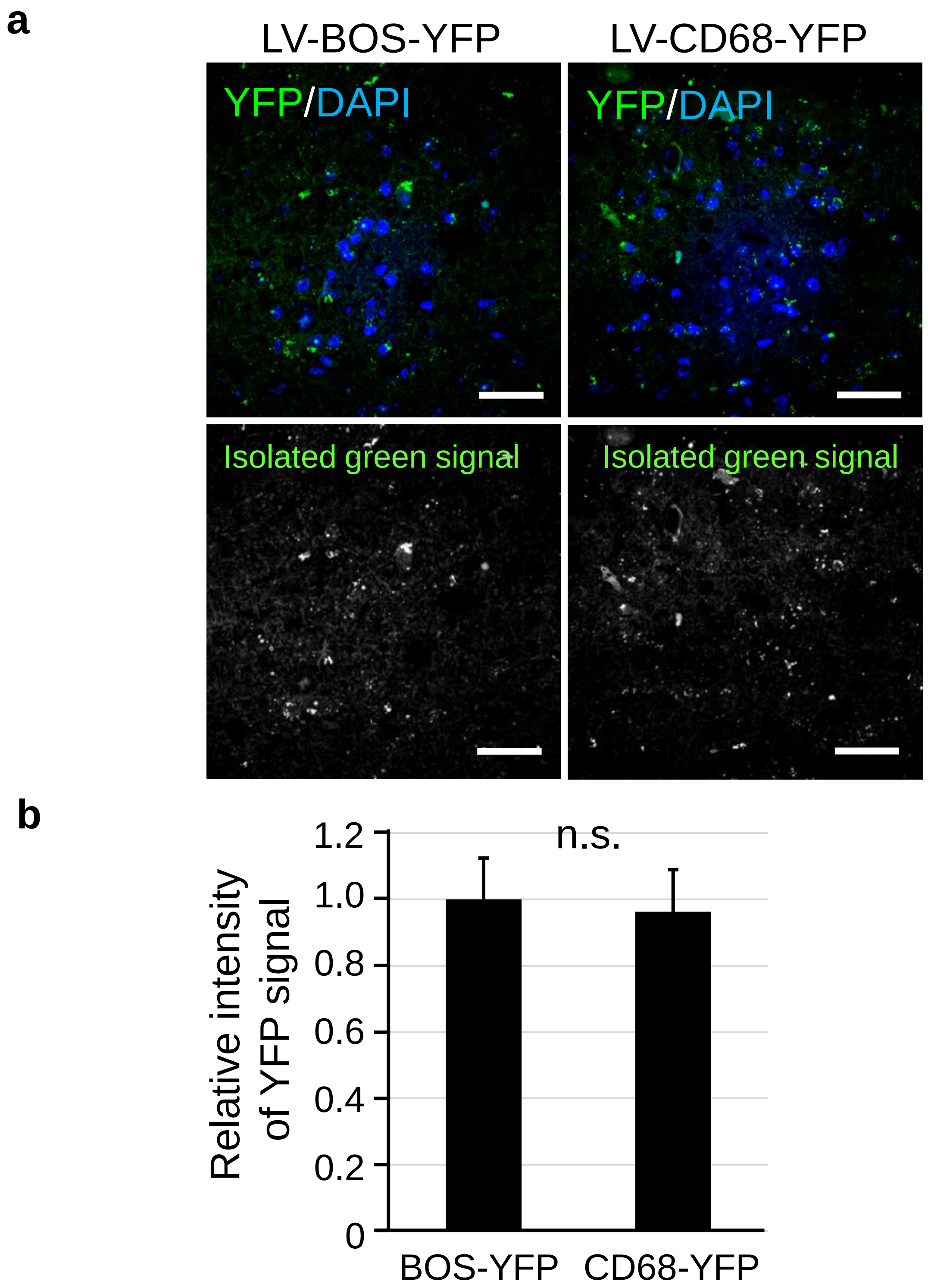

Supplementary Figure 1.
(a) YFP (green) and DAPI (blue) signals in sections of the spinal cord from SOD1(G93A) mice after bone marrow transplantation with LV-BOS-YFP or LV-CD68-YFP gene transduction. The left panel shows an image of the spinal cord in the LV-BOS-YFP vector group. The right panel shows an image of the spinal cord in the LV-CD68-YFP vector group. Upper row shows the color images and lower row shows black and white images of green color (YFP staining) isolated from the corresponding upper row. Scale bar = 50 μm.
(b) The bar graph shows relative intensity of YFP signal in same two groups (n=5 in each group). The intensity of YFP signal was measured in the black and white image in (a) using the Image J software and the ratio of relative intensity of YFP in LV-CD68-YFP group was calculated against the intensity of that in LV-BOS-YFP group. Error bars represent the mean + SD. n.s.: not significant diference.

Supplementary Figure 2

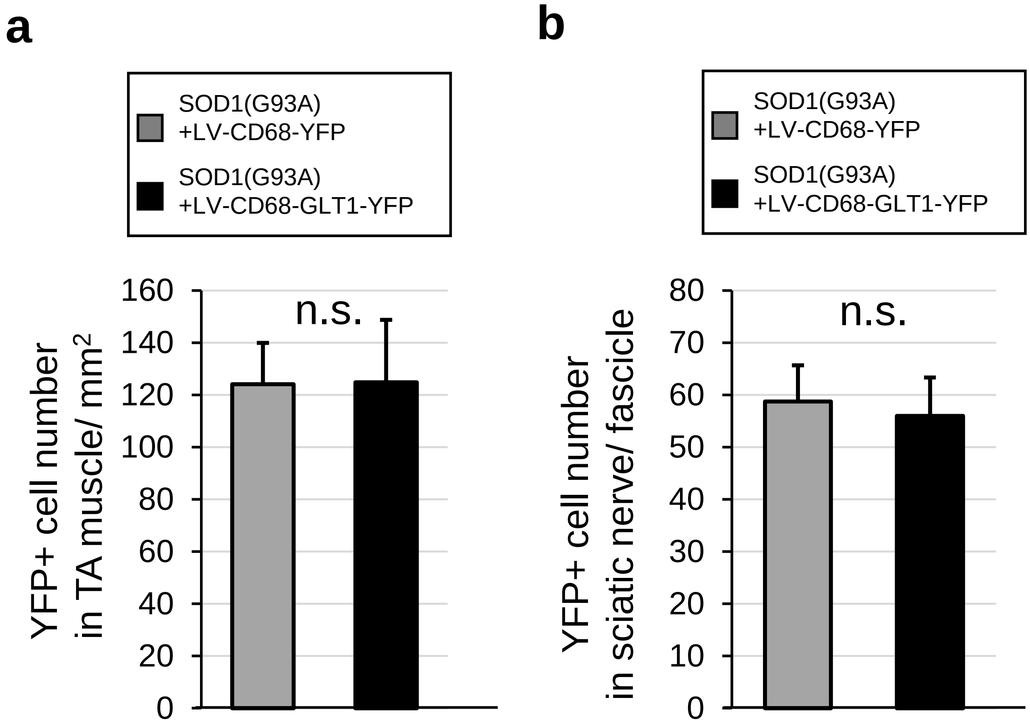

Supplementary Figure 2 .
(a) The bar graph shows the YFP positive cell number in tibialis anterior muscle in Figure 7 of SOD1(G93A) mice +LV-CD68-YFP and SOD1(G93A) mice +LV-CD68-GLT1-YFP. **(b)** The bar graph shows the YFP positive cell number in sciatic nerve per fascicle in Figure 7 of SOD1(G93A) mice +LV-CD68-YFP and SOD1(G93A) mice +LV-CD68-GLT1-YFP. Error bars represent the mean + SD. n.s.: not significant difference, TA: tibialis anterior.

Supplementary Figure 3

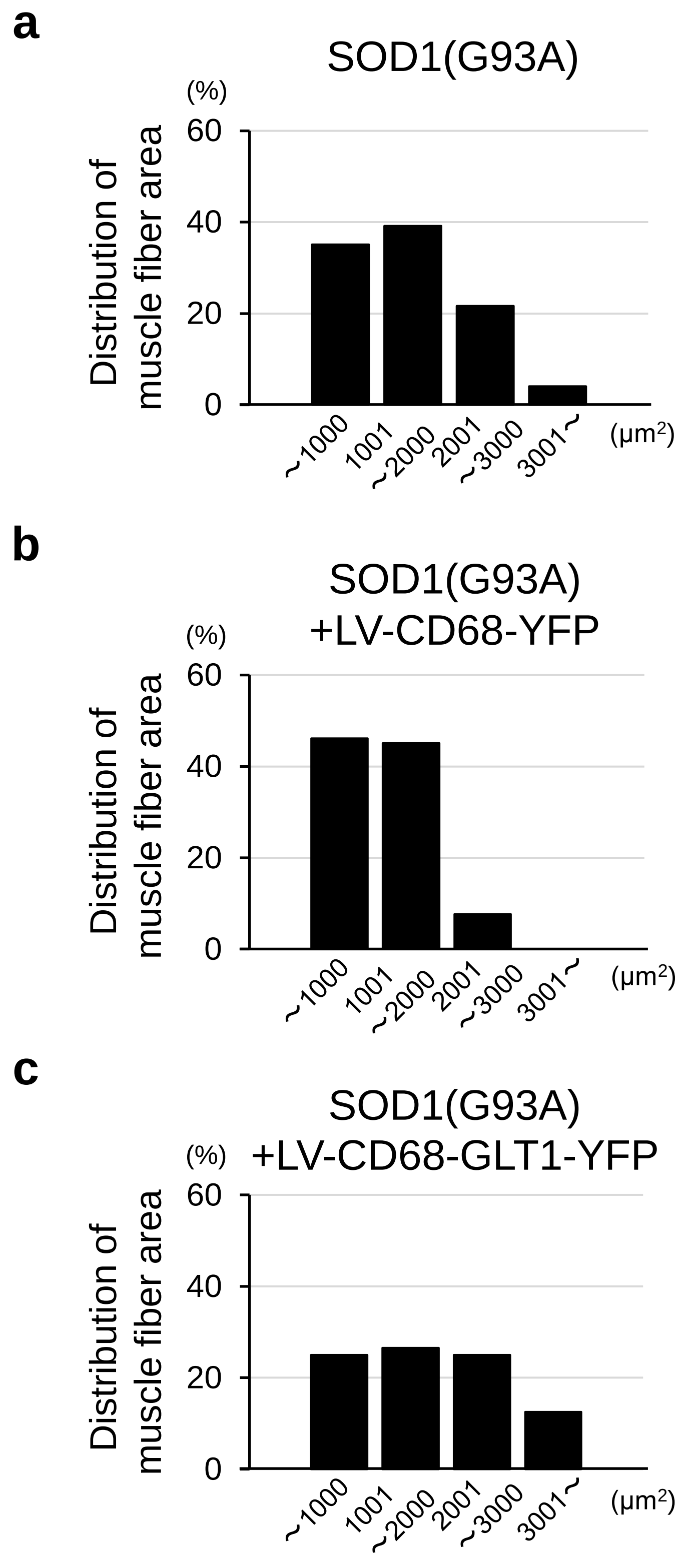

Supplementary Figure 3.
(a-c) The bar graphs show the distribution of muscle fiber area in anterior tibial muscle of **(a)** SOD1(G93A) mice, **(b)** SOD1(G93A) mice +LV-CD68-YFP and **(c)** SOD1(G93A) mice +LV-CD68-GLT1-YFP. X-axis shows the range of muscle fiber area by each 1000 μm^2 . Y-axis shows the percentage of the population in each rage of muscle fiber area.
